# Psychological status of patients with functional anorectal pain and treatment efficacy of paroxetine in alleviating the symptoms: a retrospective study

**DOI:** 10.1038/s41598-023-45401-y

**Published:** 2023-10-21

**Authors:** Mingfeng Fan, Laian Li, Xiangjun Xu, Cong Zhou, Peng Wang, Wanbin Yin, Wenju Pei, Shuai Wang

**Affiliations:** 1 Department of Anorectal Surgery, Affiliated Hospital of Jining Medical University, Jining, China; 2 Department of Psychology, Affiliated Hospital of Jining Medical University, Jining, China; 3 School of Mental Health, Jining Medical University, Jining, China

**Keywords:** Psychology, Gastroenterology, Neurology, Signs and symptoms

## Abstract

The aim of this study was to investigate the clinical characteristics, psychological status, sleep quality, and quality of life of patients with functional anorectal pain (FAP). The study also assessed the treatment efficacy of paroxetine in alleviating FAP symptoms. A retrospective comparative study of forty-three patients with FAP who were first treated with an anal plug compound glycolate suppository versus paroxetine combined with anal plug compound glycolate suppository between November 2021 and August 2022. Pain, quality of life, depression, anxiety and sleep quality were assessed before and after treatment by the Chinese version of the Short-Form McGill Pain Questionnaire-2 (SF-MPQ-2), Health-related quality of life scale (The 12-Item Short-Form Health Survey, SF-12), 17-item Hamilton Depression Rating Scale (HDRS), 14-item Hamilton Anxiety Scale (HAMA), and Pittsburgh Sleep Quality Index (PSQI). A total of 46.5% of patients with FAP were found to have anxiety symptoms (HAMA ≥ 7), 37.2% of patients with FAP were found to have depressive symptoms (HDRS ≥ 8). A total of 32.6% of patients with FAP had sleep disorders (PSQI > 10). Within 1 week after drug withdrawal, the short-term efficacy rate of oral paroxetine was 95.5%. After treatment, the symptom pain score (VAS) and sleep score were lower than those before treatment (P < 0.01). In the areas of vitality (VT), Social Functioning (SF), and Mental Health (MH), the difference between the pre-treatment and 8 weeks posttreatment scores of the study group and the control group was statistically significant (P < 0.05). FAP patients have obvious symptoms of anxiety and depression, and the incidence of sleep disturbance is prevalent. Paroxetine, a typical serotonin reuptake inhibitor (SSRI), was able to alleviate depression, anxiety, and pain symptoms in FAP, which might have clinical application prospects.

## Introduction

Functional anorectal pain (FAP) is a non-organic anorectal disorder that occurs in the anus and rectum. According to the specific pain history and clinical symptoms, the clinical diagnosis of FAP can be divided into chronic anal pain and spastic anal pain. According to the duration, frequency and signs of pain, FAP can be divided into three subtypes: spastic anorectal pain, anal levator syndrome, and nonspecific functional anorectal pain^1–6^. Persistent pain during chronic anal pain attacks can last for at least 20 min. Digital rectal examination with or without pubic rectal tenderness can distinguish levator anus syndrome from nonspecific anorectal pain. Spastic anal pain lasts from several seconds to several minutes, with no anal or rectal pain during remission intervals^2^. The prevalence of FAP has been gradually increasing in recent years, with a prevalence rate of approximately 6.6% to 11.6% worldwide, most of whom are middle-aged women^7,8^. Symptoms of FAP patients often occur repeatedly, accompanied by psychological distress, which can cause defecation disorders, depression, anxiety, and sleep problems^9,10^. Symptoms of FAP may be intermittent or continuous during the days and deteriorating in the night due to the supine position^11,12^. Moreover, functional gastrointestinal symptoms might be also associated with obesity^13^ and eating disorders^9,14^. Thus, a significant proportion of FAP patients are experiencing unsatisfactory quality of life^15–17^.

At present, many measures have been adopted to treat FAP, including biofeedback, sacral nerve stimulation and botulinum toxin therapy, drug therapy, and psychological intervention^3,18^. In addition, traditional Chinese medicine, such as warm water sitting bath, acupuncture, physiotherapy or other measures, has also been widely used^15,19–21^. Mood disorders, including depression and anxiety, are common health issues^22^. Pain and mood disorders frequently co-occur^17^, and chronic pain significantly increases the probability of mood disorders^23^. The combination of analgesics, psychotherapy and antidepressants can significantly relieve the symptoms of patients with chronic pain^16,23–25^. Paroxetine is a typical serotonin-reuptake inhibitor (SSRI) used to treat a variety of mental health conditions including depression, anxiety disorders, obsessive–compulsive disorder, and post-traumatic stress disorder. It works by inhibiting the reuptake of serotonin in the brain, thereby increasing serotonin levels and improving mood^26^. A large number of clinical studies have found that paroxetine is superior to placebo therapy and cognitive behavioral therapy in treating major depressive disorder and anxiety disorders^27^. Patients with depression and anxiety often suffer from stigma^28,29^. Therefore, FAP patients with emotional disturbances often don't disclose their mental condition to non-mental health professionals^9^. Thus, clinicians, especially surgeons, usually ignore the psychological status, sleep quality and quality of life of FAP patients, which may underlie the poor clinical treatment effect of this disease.

In this research, our first purpose was to analyze the psychological manifestations, including depression and anxiety symptoms, sleep quality, and quality of life, of FAP patients. Second, this study aimed to retrospectively analyze the psychological status of FAP patients after paroxetine treatment to provide a reference for the future clinical treatment of FAP.

## Materials and methods

### Participants

From November 2021 to August 2022, patients with FAP were recruited to the outpatient and inpatient departments of the Anorectal Department of the Affiliated Hospital of Jining Medical University. Informed consent was obtained from all the individual patients in this study. The Affiliated Hospital of Jining Medical College Ethics Committee approved this study (approval number: 2023-04-C020). A total of 50 patients with FAP were prospectively recorded, and the database was established. The data of 43 patients who finally met the inclusion criteria of this study were analyzed retrospectively.

The inclusion criteria were as follows: (1) meet the diagnostic criteria of functional anorectal pain in Rome IV of functional gastrointestinal diseases; (2) patients treated with paroxetine combined compound carragonium suppositories (study group) or treated with compound carragonium suppositories only (control group) for at least 4 weeks; and (3) complete data records. The exclusion criteria were as follows: (1) patients with severe cardiac insufficiency, liver insufficiency, renal insufficiency, or nervous system diseases; (2) those who did not complete effective treatment (self-withdrawal or nonmedication); (3) anorectal pain induced by other causes, such as inflammatory bowel disease, anal fistula, abscess, hemorrhoids, anal fissure and other organic diseases; and (4) the presence of other anorectal organic pathology was confirmed by colonoscopy or anorectal MRI.

### Treatment and methods

All patients with FAP received regular health education and psychological counseling, completed by an experienced anorectal clinician at least 3 times, including explaining the relevant knowledge of FAP, explaining the etiology, guiding diet and self-care, explaining the relationship between emotion and illness, and adjusting for negative emotion. All patients with FAP were treated with a warm water sitting bath for 10–15 min, and compound carragonium suppositories were embolized into the anus 2–3 cm, one tablet at a time, once a day. The drugs were used continuously for 4 weeks. Among the 43 FAP patients who met the inclusion criteria, 22 FAP patients agreed to and received paroxetine hydrochloride tablets were assigned to the study group.

The general data questionnaire was collected, and clinical symptom ratings were evaluated before, one week, and 4 weeks after treatment. The above evaluations were assessed by one experienced clinical psychiatrist. Our study excluded patients with mental illness by one experienced clinical psychiatrist. All methods were carried out in accordance with relevant guidelines and regulations.

The 17-item Hamilton Depression Rating Scale (HDRS, Cronbach alpha: 0.714)^30^ and 14-item Hamilton Anxiety Scale (HAMA, Cronbach alpha: 0.74)^31^ were used to evaluate the symptoms of depression and anxiety and to reflect the psychological characteristics of the disease. Compared with self-assessment scales such as the Patient Health Questionnaire Depression (PHQ-9) and Generalized Anxiety Disorder Questionnaire (GAD-7), the HDRS and HAMA are more professional and authoritative and have better internal consistencies. The total score indicates different levels of depressive or anxious symptoms: HDRS scores < 8 indicate normal, scores from 8 to 17 indicate potential depression, scores from 17 to 35 indicate definite depression, and a total score > 35 indicates severe depression. A total HAMA score < 7 points was considered normal, scores from 7 to 14 indicate possible anxiety, scores from 14 to 21 indicate definite anxiety, scores from 21 to 29 indicate obvious anxiety, and scores > 29 indicate severe anxiety. The Pittsburgh Sleep Quality Index (PSQI, Cronbach alpha: 0.82–0.83)^9,32,33^ assesses the sleep quality of the subjects in the last month. The PSQI is useful in psychiatric clinical practice and research activities. Sleep quality scores from 0 to 5 indicate very good sleep quality, scores from 6 to 10 indicate good sleep quality, s scores from 11 to 15 indicate average sleep quality, and scores from 16 to 21 indicate very poor sleep quality. The higher the PSQI score is, the worse the sleep quality. Quality of life was evaluated with the 12-Item Short-Form Health Survey scale (SF-12, Cronbach alpha: 0.63–0.91)^34^. The higher the total score, the better the quality of life. It is a simplified version of the SF-36, with a total of 12 items and 8 dimensions for evaluating health-related quality of life, namely, physical functioning (PF), role physical (RP), bodily pain (BP), vitality (VT), social functioning (SF), role emotional (RE), and mental health (MH). With the advantages of being simple, short, easy to understand and requiring less operation time, it is easy for research subjects to accept, and high-quality data can be obtained^34^.

### Evaluation of curative effect

The visual analog scale (VAS) was used to evaluate current pain. A score of 0 indicate no pain, scores from 1 to 3 indicate mild pain, scores from 4 to 6 indicate moderate pain, and scores from 7 to 10 indicate severe pain, unbearable appetite and sleep. At baseline, all patients were evaluated by the Chinese version of the Short-Form McGill Pain Questionnaire-2 (SF-MPQ-2, Cronbach alpha: 0.73–0.95)^35^ as well as the VAS. Patients assessed the degree of swelling or other symptoms on a visual analog scale ranging from 0 to 10^9^. The patients were evaluated and recorded again after four weeks of treatment.

According to the evaluation standard of functional anorectal disease Rome IV, the curative effect was evaluated by the score reduction rate of the simple pain questionnaire. Specific methods: (total score before treatment—total score after treatment)/total score before treatment × 100%. Cure: symptom score is reduced by more than 90%; markedly effective: symptom score is reduced by 70–90%; effective: symptom score is reduced by 30%-70%; ineffective: symptom score is reduced by less than 30%. Effective rate = (cured + markedly effective + effective) cases/total cases × 100%.

### Statistical analysis

Data were analyzed with IBM SPSS version 26. All measurements are presented as the mean ± standard deviation. Measurement data were compared by *t* tests, paired *t* tests were to compare pre- and posttreatment data, and two-sample* t* tests were used for comparisons between groups. Count data were compared by the chi-squared test, and *P* < 0.05 was considered statistically significant. Pre- and posttreatment assessments were conducted at baseline and after 4 weeks and 8 weeks. HDRS, HAMA, PSQI, and SF-MPQ-2 scores were evaluated using nonparametric tests, paired samples nonparametric tests within groups, and independent samples nonparametric tests between groups, and these data were not normally distributed. The Spearman correlation coefficient (r) was conducted to assess the linear relationship between SF-MPQ-2, HDRS, HAMA, PSQI, and the progression of the disease.

## Results

**Figure 1:**
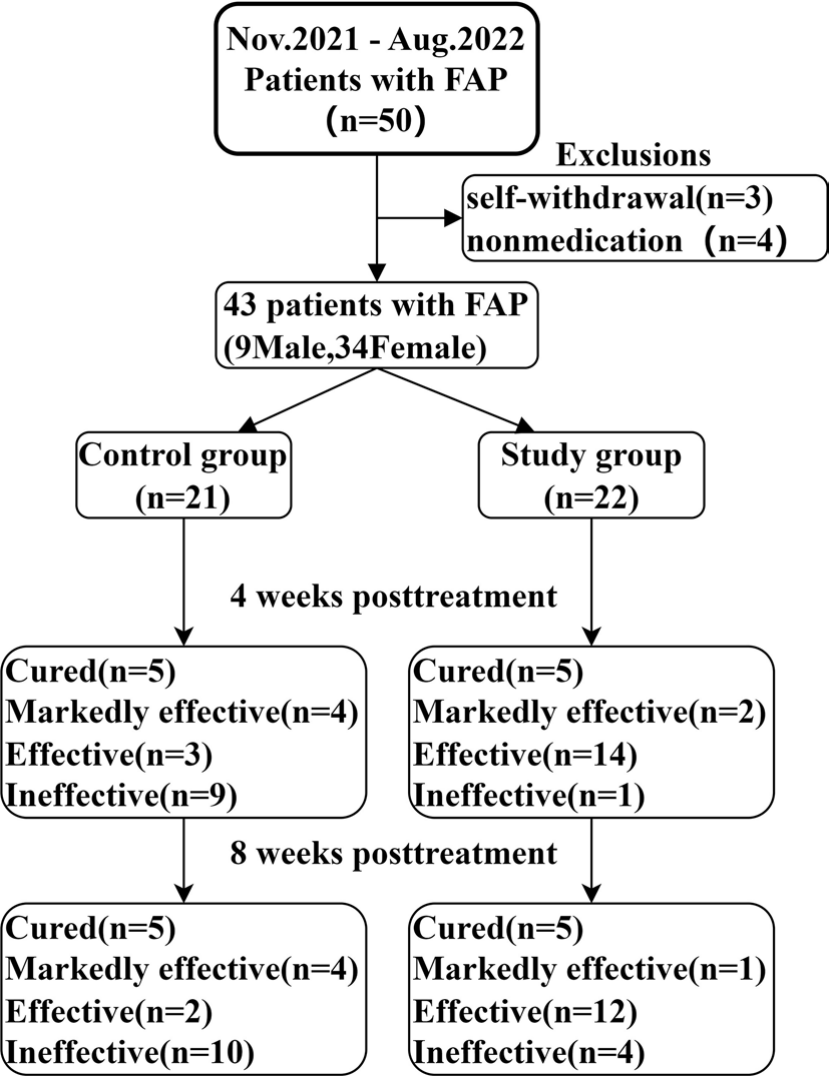
The treatment of FAP patients, and the follow-up treatment effect at 4 weeks and 8 weeks post-treatment.

A total of 50 patients with FAP were identified from November 2021 to August 2022 (Fig. 1). Of the 7 excluded patients, 3 were excluded because of self-withdrawal and 4 were excluded because of non-medication. Of the 43 included patients, 21 underwent control group and 22 underwent study group.

**Figure 2:**
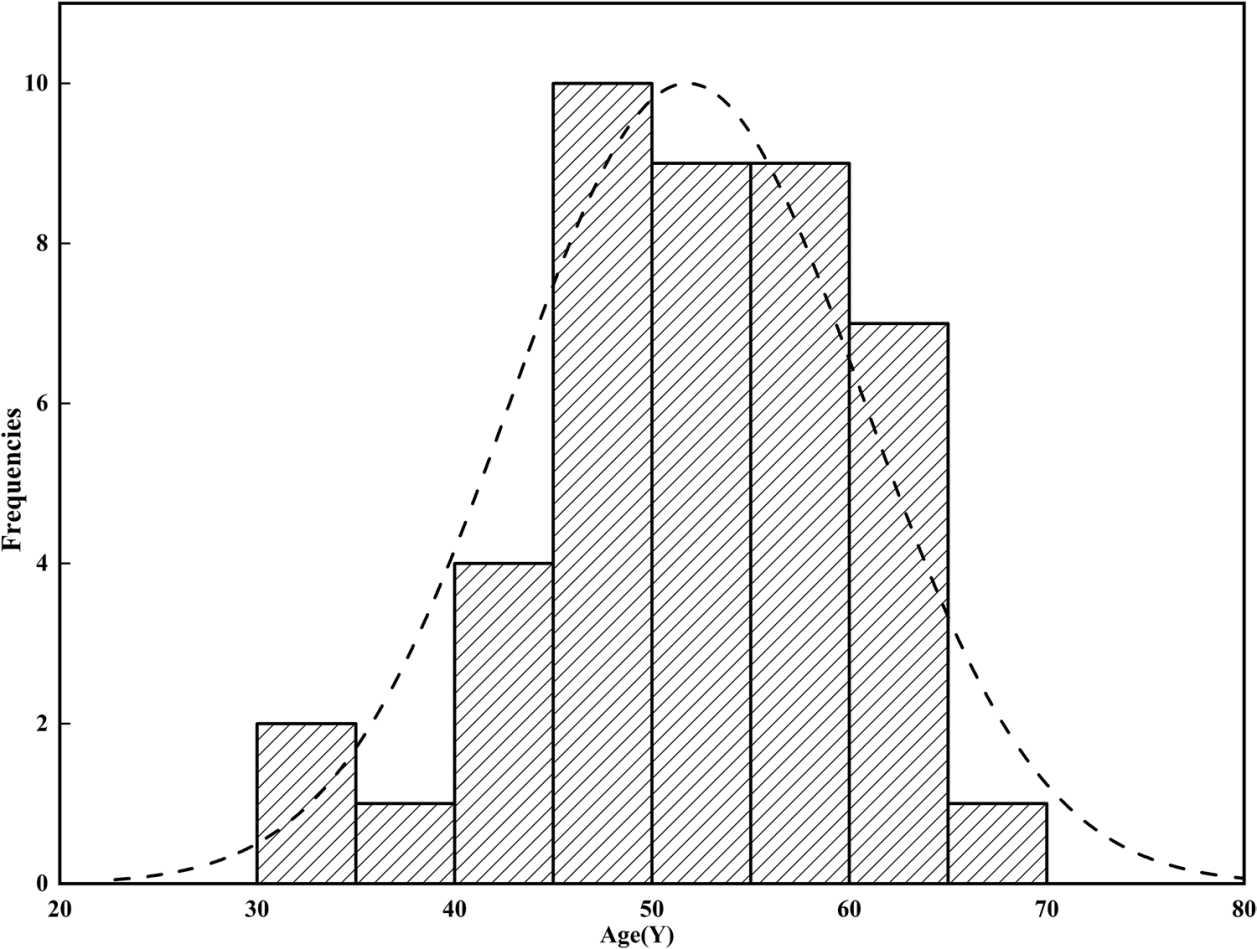
The age distribution of all FAP patients, which was approximately normal.

**Figure 3:**
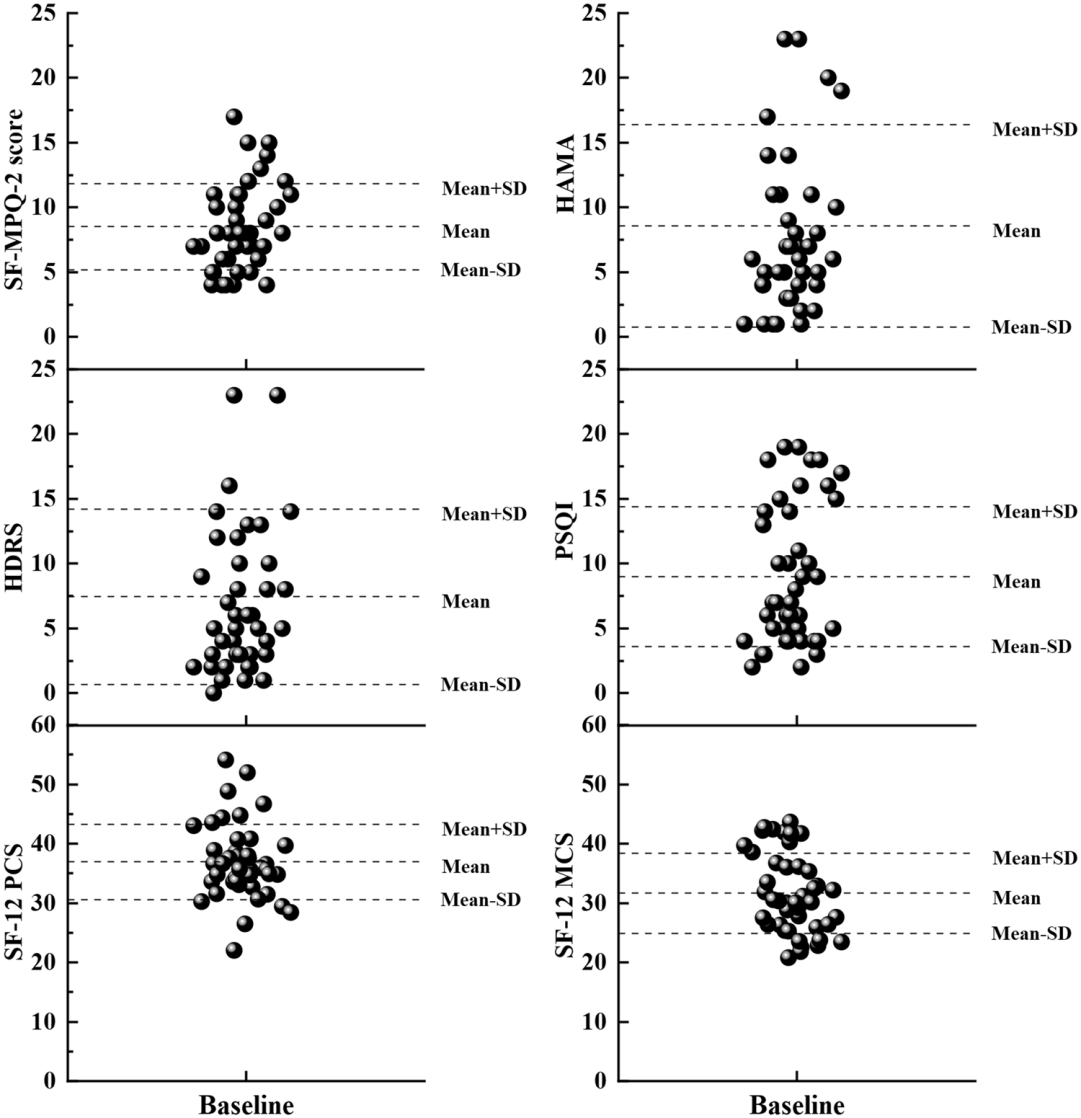
The scatter plots of HDRS, HAMA, SF-MPQ-2, PSQI, SF-12 PCS, SF-12 MCS scores of FAP patients at baseline. Means and standard deviations are described.

Table 1 shows the age, sex, course of the disease, pain characteristics, and other symptoms of the FAP patients in detail. Figure 2 shows the age distribution of all FAP patients, which was approximately normal. Of all the patients, 34 patients (79.1%) were female. A total of 74.4% of the patients had definite pain, of which 65.1% described the pain as falling pain, and 9.3% described the pain as soreness. In addition to pain, anal drooping and distension sensation and an inexhaustible sense of defecation are more frequent. According to the psychometric instruments, a total of 37.2% of patients showed depressive symptoms (HDRS ≥ 8). A total of 44.2% of patients with FAP showed anxiety symptoms (HAMA ≥ 7). A total of 32.6% of the patients had sleep disturbance (PSQI > 10). Figure 3 shows the scatter plots of SF-MPQ-2, HDRS, HAMA, PSQI, SF-12 PCS, SF-12 MCS scores among FAP patients at baseline. The depression, anxiety, and sleep symptom severities were mutually correlated with each other, and all of them were significantly correlated with pain symptoms (Table 2).

**Table 1:** Demographic characteristics, clinical symptoms, and pain characteristics of FAP patients (N=43).

Characteristics	N (%)/M ± SD	LAS (n = 18)	UFAP (n = 21)	PF (n = 4)
Grouping				
Control group	21	8	10	3
Study group	22	10	11	1
Age (years)				
< 40	3 (7.0%)			
40–60	32 (74.4%)			
> 60	8 (18.6%)			
Gender				
Male	9 (20.9%)	5	4	0
Female	34 (79.1%)	13	17	4
Disease course (years)				
0.5–1	27 (62.8%)			
1–5	13 (30.2%)			
5–10	3 (7%)			
Symptom				
Pain	32 (74.4%)	13	15	4
Anal drooping and distension sensation	28 (65.1%)	13	12	3
Inexhaustible sense of defecation	24	10	11	3
Burning sensation of the anus	5	3	2	0
Anorectal obstruction sensation	6	4	2	0
Itching sensation	2	0	2	0
Anorectal crawling sensation	2	1	1	0
Anal foreign body sensation	1	1	0	0
Anal dampness	1	1	0	0
Pain characteristics				
Distending pain	28 (65.1%)	12	14	2
Sore pain	4 (9.3%)	3	1	0
Burning pain	1	0	1	0
Spasmodic pain	2	1	0	1
Tingling pain	1	0	0	1
Throbbing pain	1	1	0	0
Radiation pain	1	0	1	0
Sleep disturbance				
Yes	14 (32.6%)			
No	29 (67.4%)			
VAS	1.91 ± 0.72			
HDRS	7.44 ± 6.78			
HAMA	8.58 ± 7.80

**Table 2:** Spearman correlation coefficient and *P*-value matrix between SF-MPQ-2, HDRS, HAMA, PSQI, and disease course.

	SF-MPQ-2		HDRS		HAMA		PSQI	
	r	*P*	r	*P*	r	*P*	r	*P*
SF-MPQ-2	1		0.545	< 0.001***	0.426	0.004**	0.496	0.001**
HDRS	0.545	< 0.001***	1		0.816	< 0.001***	0.541	< 0.001***
HAMA	0.426	0.004**	0.816	< 0.001***	1		0.618	< 0.001***
PSQI	0.496	0.001**	0.541	< 0.001***	0.618	< 0.001***	1	
Disease course (Months)	− 0.033	0.834	− 0.300	0.050	− 0.265	0.086	− 0.225	0.147

*P < 0.05, **P < 0.01, ***P < 0.001.

There was no significant difference in age, sex, or disease course between the two groups (*P* = 0.154,* P* = 0.767,* P* = 0.987). The total SF-MPQ-2 score, sensory pain score, emotional pain score, and VAS score were statistically analyzed. At 4- and 8-weeks posttreatment, the pain VAS score of patients in the study group was lower than that before treatment (2.00 ± 0.69 vs. 0.68 ± 0.72 *P* < 0.001; 2.00 ± 0.69 vs. 0.86 ± 0.94,* P* < 0.001). At 4 weeks posttreatment, the effective rate of the control group with anal plug compound glycolate suppository was 57.1%, while that of the study group with paroxetine treatment was 95.5%. There was a significant difference in the efficacy rate between the two groups (*P* = 0.003). At 8 weeks posttreatment, there was still a significant difference in the efficacy rate between the study and control groups (81.8% vs. 52.4%, *P* = 0.039) (Fig. 1, Table 3).

**Table 3:** The comparison of age, sex, course of disease and effective rate between the control group and the study group.

Grouping	Age (years)	Gender (male/female)	Disease course (months)	Effective rate (4 weeks)	Effective rate (8 weeks)
Control group	49.76 ± 9.87	4/17	16.29 ± 16.77	57.1%	52.4%
Study group	53.68 ± 7.74	5/17	16.18 ± 25.11	95.5%	81.8%
*P* value	0.154^a^	0.767^b^	0.987^a^	0.003^b^	0.039^b^

^a^The *P* value represents the result of a two-sample t-test.

^b^The *P* value represents the result of a chi-squared test.

**Figure 4:**
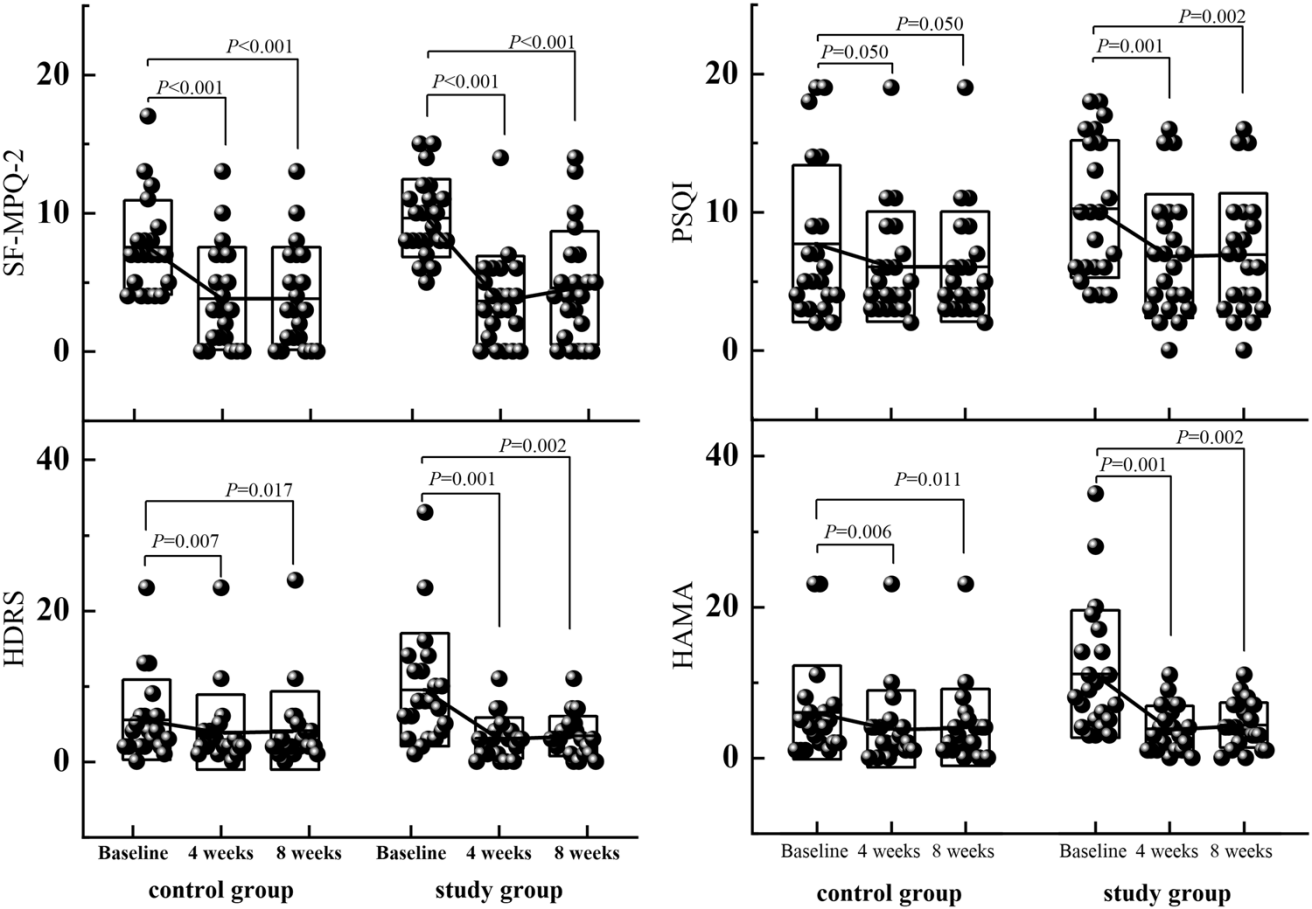
The box plot of scores and the folded graphs of the mean HDRS, HAMA, SF-MPQ-2, PSQI scores at baseline, and 4, 8 weeks posttreatment for both groups.

There were significant differences in the study group's HDRS score and HAMA score pre- and post-treatment. There was no significant difference in sleep quality score between the two groups’ pretreatment (*P* = 0.129), but there was a significant difference in the study group pre- and posttreatment (*P* = 0.001, *P* = 0.002). In these FAP patients, paroxetine effectively relieved pain symptoms, alleviated depressive symptoms, and anxiety symptoms, and improved sleep quality (Fig. 4). Figure 4 shows the box plot of scores and the folded graphs of the mean scores on the SF-MPQ-2, PSQI, HDRS, HAMA at baseline and 4- and 8-weeks posttreatment for both groups.

**Figure 5:**
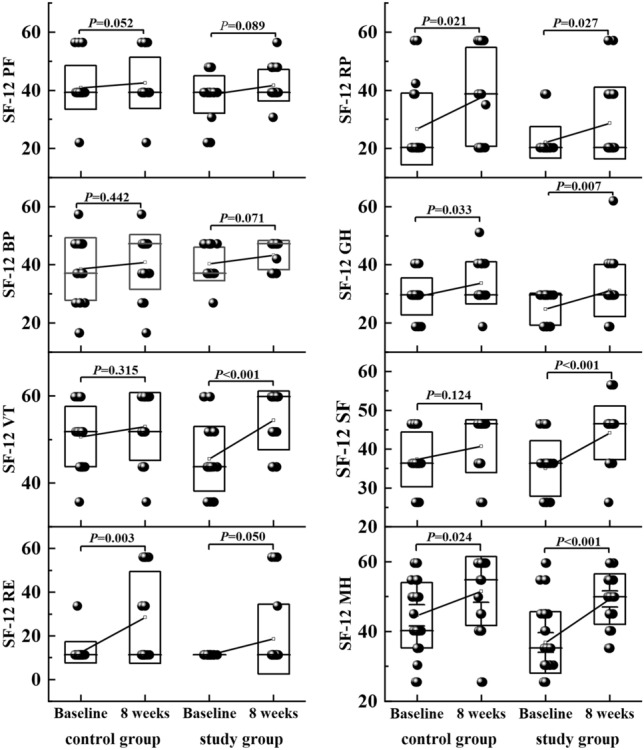
The box plot of SF-12 eight dimensions at baseline, 8 weeks posttreatment for both groups. Means, standard deviations and median are described. *PF* physical functioning, *RP* role physical, *BP* bodily pain, *VT* vitality, *SF* social functioning, *RE* role emotional, *MH* mental health.

The scores of all 8 dimensions of the SF-12 scale were lower than normal. Following treatment, patients demonstrated improved scores across all dimensions, with statistically significant differences observed between pretreatment and 8 weeks posttreatment scores, except for PF and BP, among the study group. In the areas of VT, SF, and MH, the difference between the pre-treatment and 8 weeks posttreatment scores of the study group and the control group was statistically significant (Fig. 5).

## Discussion

In this current study, using HAMA and HDRS screening, 46.5% of patients with FAP were found to have possible anxiety symptoms (HAMA ≥ 7), 37.2% of patients with FAP had possible depressive symptoms (HDRS ≥ 8). The severity of anxiety, depression, and sleep symptoms is correlated in pairs, and all of them were significantly correlated with pain symptoms. These findings indicate that depressive and anxiety symptoms were quite common among patients with FAP. One recent study reported that the prevalence of anxiety and depression was 46% and 55%, respectively, using PHQ-9 and GAD-7 screening^9^. 67.4% of patients with FAP had good sleep quality (PSQI ≤ 10). Our results confirmed the above-mentioned discoveries, and indicated chronic pain and mood disorders may be closely interrelated in a bidirectional manner^16^.

Previous research indicated that the pain of FAP mainly located in the anal canal, rather than the rectum^24^. Our findings suggested that some patients with FAP experience symptoms of distension instead of pain, while others report more frequent pain in the anal canal. These symptoms bring heavy disturbances to the quality of life of the FAP patients, emphasizing the importance of accurate diagnosis and effective clinical treatment of this disease^2,18,36,37^. Short-term effects such as biofeedback, sacral nerve stimulation, pudendal nerve block, drugs, and psychotherapy have shown positive results, while surgical treatment was often ineffective^15,19,38,39^. Some studies have suggested that the pathogenesis of FAP involves abnormal processing of pain signals in the central nervous system, and some initiating factors lead to anal and rectal hypersensitivity. Holistic approaches such as hypnosis, biofeedback, acupuncture, and psychotherapy, may enable some patients understand the psychological dynamic factors that cause pain and may alleviate the symptoms^19,24,40^. Patients with FAP suffered from comorbid mood disorders, including depression, anxiety, and sleep disorders revealed by our study, which was consistent with previous findings^9,10^. The intercorrelation of depression, anxiety, and sleep symptom severities, all significantly linked to pain symptoms, highlights the considerable distress that patients might experience, adding to their overall life burden. Therefore, we investigated paroxetine as a special therapy to improve the patient's mood condition, and tried to eliminate the anorectal pain symptoms. Paroxetine as a typical SSRI, has been approved indications for the treatment of depression, obsessive–compulsive disorder, panic disorder and social phobia. It is also used in the treatment of generalized anxiety disorder, posttraumatic stress disorder, premenstrual dysphoric disorder and chronic headache^27,41^. Specially, paroxetine has distinct therapeutic effects on chronic pain, as well as gastrointestinal discomfort symptoms with psychiatric comorbidity^42,43^. In our present research, in the study group using paroxetine, the symptoms of anorectal pain were significantly alleviated after treatment, which was manifested by the improvement of the SF-MPQ-2 scale score, with an effective rate of 95.5%. In comparison, the effective rate of the control group was 57.1%. The difference between the two groups was statistically significant (*P* < 0.01). Compared to single use of compound carragonium suppositories, paroxetine not only significantly improved somatic symptoms, but also alleviated anxiety and depressive mood, thereby improving the overall quality of life of FAP patients. With the supplement of paroxetine as an antidepressant medication, FAP patients would benefit more from the comprehensive treatment. Our findings could provide novel perspectives on the underlying pathological mechanisms and personalized treatments for psychosomatic disorders in the future.

The comorbidity of insomnia and pain is highly prevalent^44^, and might share common genetic basis^45^. Insomnia is an independent risk factor of depression, anxiety, and other mental disorders^46^. Co-occurring pain and emotional distress are associated with negative impacts on health and quality of life. These might lead to poorer treatments for both pain and mood disorders, increased economic burden, as well as elevated morbidity and mortality rates^17^. Although paroxetine does not have a direct effect on sleep, our results have shown that the sleep quality of the study group has also improved with the alleviation of depressive and anxiety symptoms. Attentions should be paid to the mood and sleep problems of patients with FAP by doctors, as psychological issues could increase the burden of disease of those suffering from FAP. Moreover, mental disorders are known to influence doctor-patient relationship and adherence to therapy^47^. Thus, it is crucial for psychologists to be involved in the treatment of patients with FAP^9^.

In our present research, in the study group using paroxetine, the symptoms of anorectal pain were significantly alleviated after treatment, which was manifested by the improvement of the SF-MPQ-2 scale score, with an effective rate of 95.5%. In comparison, the effective rate of the control group was 57.1%. We found significant improvements in the domains of vitality (VT), social functioning (SF), and mental health (MH). These improvements are particularly noteworthy as they reflect the psychosocial aspects of quality of life. Patients in the study group reported increased vitality, indicating a higher level of energy and enthusiasm. Furthermore, their social functioning and mental health showed significant enhancement, suggesting improved interpersonal relationships and emotional well-being. The quality-of-life assessment is crucial to understanding the impact of pain on patients' well-being and can serve as a valuable tool for monitoring treatment response. For the treatment of FPA, relieving pain symptoms is only one aspect of the process. Our ultimate aim is for patients to return to society as soon as possible and regain their normal work and life abilities. Based on our findings, short-term paroxetine treatment for FAP not only alleviated symptoms of anorectal discomfort, but also relieved the patients' emotional problems, and finally significantly improved their quality of life. This is of great significance for the complete recovery of the FAP patients. However, it's important to note that not all domains of quality of life demonstrated significant changes. In our study, we did not observe significant improvements in the physical functioning (PF) and bodily pain (BP) domains. This could be attributed to the fact that our treatment primarily targeted psychological symptoms and anorectal discomfort, while the physical aspects of FAP may require a different approach or longer-term interventions.

There are several limitations to this study. First, the cross-sectional design can only demonstrate associations between outcome variables and emotional disorders, including depression, anxiety disorders, and sleep disorders, and no causal inferences can be made among the studied outcome variables. Since the duration of treatment for paroxetine (SSRI) to be effective is usually longer than 4 weeks, thereby indicating the need for longer follow-up periods in future research. Future prospective studies that extend the follow-up period will be helpful in the discussion and elucidation of the causal relationship between treatment and outcome. Second, our study was conducted in a single center with a limited sample size, which might influence the generalizability of these findings. Multicenter studies with large samples are needed to verify our findings. Third, the use of paroxetine in this current study was somewhat off-label, as SSRIs such as paroxetine were typically treated for moderate to severe depressive disorders, and may have adverse side effects when prescribed off-label, including inducing suicidal thoughts. It is important to note that psychotherapy remains the first-line treatment for psychosomatic disorders. Fourth, clinical interviews based on DSM-5TR criteria are considered the gold standard for evaluating mental health. Clinical interviews allow for a more in-depth exploration of a patient's psychological well-being, offering a qualitative dimension to our findings. They enable clinicians to gather additional information, including the patient's personal history, context, and nuances of their mental health condition. Fifth, the randomization and blinding were lacking limited by the retrospective nature of the study. Last but not least, the use of psychometric tests might not be as rigorous as clinical interviews and may not fully capture the complexity of these disorders.

## Conclusion

Patients with FAP could benefit from short-term oral treatment with paroxetine, which would significantly relieve FAP symptoms and improve psychological status, sleep quality, and quality of life. We hope that our study provides valuable insights into the potential use of paroxetine as a first-line treatment option, especially for FAP patients presenting with depression and anxiety symptoms. Understanding the multifaceted benefits of paroxetine could guide clinicians in providing more comprehensive care to individuals with functional anorectal pain (Supplementary Information S1).

### Supplementary Information

Supplementary Information.

## Data Availability

All data analysed during this study are included in this published article and its supplementary information files.
